# Supplementary material for: SNHG1 knockdown upregulates miR-376a and downregulates FOXK1/Snail axis to prevent tumor growth and metastasis in HCC
Source: Mol Ther Oncolytics. 2021 Feb 4;21:264–77. doi: 10.1016/j.omto.2021.02.002 (PMC8143978; doi:10.1016/j.omto.2021.02.002)
Supplement: Document S1. Figures S1–S3 [file mmc1.pdf]

OMTO, Volume *21*

Supplemental information

SNHG1 knockdown upregulates miR-376a and downregulates FOXK1/Snail axis to prevent tumor growth and metastasis in HCC

Fanzhi Meng, Jinghua Liu, Tao Lu, Lanlan Zang, Jing Wang, Qiang He, and Aijin Zhou

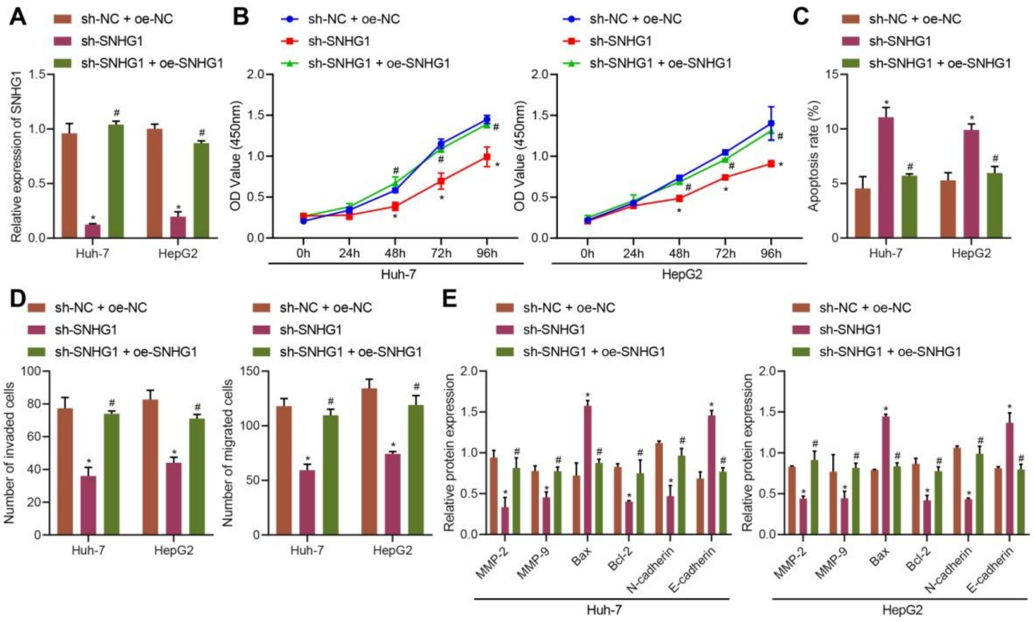

Supplementary Figure 1 Effect of downregulated SNHG1 on HCC cell phenotypes is rescued by overexpressed SNHG1. A: SNHG1 expression determined by RT-qPCR; B: Cell viability assessed by CCK8 assay; C: Cell apoptosis determined by flow cytometry; D: Cell invasion and migration determined by Transwell assay; E: Expression of MMP-2, MMP-9, Bax, Bcl-2, E-cadherin and N-cadherin proteins measured by Western blot assay; * $p < 0.05$ *vs*. cells co-transfected with sh-NC and oe-NC; # $p < 0.05$ *vs*. cells co-transfected with sh-SNHG1. Data were expressed as mean ± standard deviation. Data between two groups were compared with unpaired *t* test. Data among multiple groups were compared with one-way ANOVA with Tukey's post hoc test. Data comparison among groups at different time points was performed using repeated measures ANOVA and Bonferroni post hoc test. Each experiment was repeated 3 times independently.

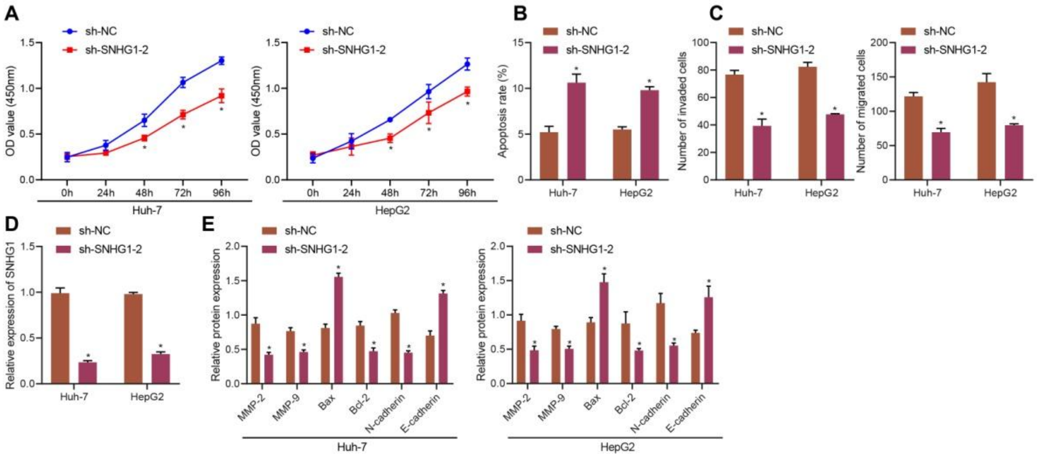

Supplementary Figure 2 sh-SNHG1-2 effectively reduces SNHG1 expression to mediate HCC cell phenotypes and related protein expression. A: Cell viability assessed by CCK8 assay; B: Cell apoptosis determined by flow cytometry; C: Cell invasion and migration determined by Transwell assay; D: SNHG1 expression determined by RT-qPCR; E: Expression of MMP-2, MMP-9, Bax, Bcl-2, E-cadherin and N-cadherin proteins measured by Western blot assay; * $p < 0.05$ *vs*. cells transfected with sh-NC. Data were expressed as mean ± standard deviation. Data between two groups were compared with unpaired *t* test. Data among multiple groups were compared with one-way ANOVA with Tukey's post hoc test. Data comparison among groups at different time points was performed using repeated measures ANOVA and Bonferroni post hoc test. Each experiment was repeated 3 times independently.

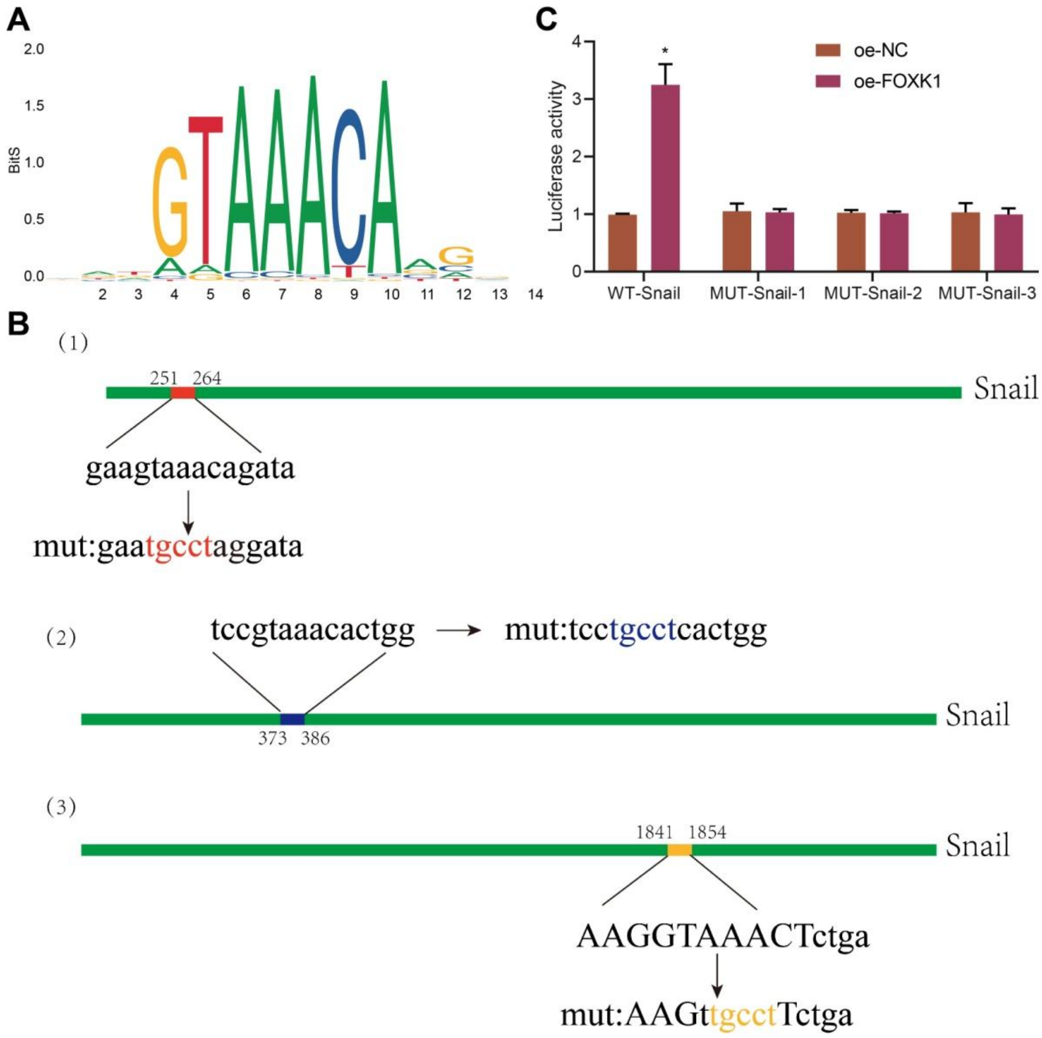

Supplementary Figure 3 Prediction results of binding sites between FOXK1 and Snail promoter region. A: Matrix model for prediction on binding motifs between FOXK1 and Snail; B: Binding sites between FOXK1 and Snail and the corresponding mutation.
