# Supplementary material for: SNHG1 knockdown upregulates miR-376a and downregulates FOXK1/Snail axis to prevent tumor growth and metastasis in HCC
Source: Mol Ther Oncolytics. 2021 Feb 4;21:264–77. doi: 10.1016/j.omto.2021.02.002 (PMC8143978; doi:10.1016/j.omto.2021.02.002)
Supplement: Document S2. Article plus supplemental information [file mmc2.pdf]

Molecular Therapy
Oncolytics
Original Article

SNHG1 knockdown upregulates miR-376a and downregulates FOXK1/Snail axis to prevent tumor growth and metastasis in HCC

Fanzhi Meng,[1,2] Jinghua Liu,[1,2] Tao Lu,[3] Lanlan Zang,[4] Jing Wang,[5] Qiang He,[1,2] and Aijin Zhou[6]

[1]Department of Hepatobiliary Surgery, Linyi People's Hospital, Linyi 276000, P.R. China; [2]Prof. Dr. Cai's Laboratory, Linyi People's Hospital, Linyi 276000, P.R. China; [3]Shandong Coal Linyi Hot Spring Sanatorium, Linyi 276000, P.R. China; [4]Central Laboratory, Linyi People's Hospital, Linyi 276000, P.R. China; [5]Department of Radiology, Linyi People's Hospital, Linyi 276000, P.R. China; [6]Department of Emergency, Linyi People's Hospital, Linyi 276000, P.R. China

Long non-coding RNAs (lncRNAs), microRNAs (miRNAs or miRs), and genes are emerging players in cancer progression. In the present study, we explored the roles and interactions of oncogenic lncRNA small nucleolar RNA host gene 1 (SNHG1), miR-376, forkhead box protein K1 (FOXK1), and Snail in hepatocellular carcinoma (HCC). Expression of SNHG1, miR-376, and FOXK1 in HCC was characterized in clinical HCC tissues of 75 patients with HCC. The interactions between SNHG1 and miR-376 and between miR-376 and FOXK1 were predicted and confirmed by dual-luciferase reporter gene and RNA immunoprecipitation assays. Overexpression and knockdown experiments were performed in HCC cells to examine the effects of the SNHG1/miR-376/FOXK1/Snail axis on viability, apoptosis, invasiveness, and migrating abilities. Their effects on tumor growth and metastasis were validated in nude mouse models. SNHG1 and FOXK1 were upregulated, and miR-376a was downregulated in HCC. SNHG1 knockdown contributed to suppression of HCC cell viability, invasion, and migration properties and promotion of apoptosis. SNHG1 could competitively bind to miR-376a to upregulate its target gene FOXK1, which upregulated Snail. SNHG1 knockdown delayed cancer progression both *in vitro* and *in vivo* by upregulating miR-376a and downregulating FOXK1 and Snail. SNHG1 knockdown exerts anti-tumor activity in HCC, suggesting a therapeutic target.

INTRODUCTION

It has been estimated that liver cancer is the fourth most deadly cancer in the world in 2018.[1] Hepatocellular carcinoma (HCC) is the most frequent type of liver cancer. Many risk factors have been identified for this malignancy, including exposure to dietary toxins, hepatitis B and C infection, fatty liver disease, and heavy alcohol consumption.[2,3] Although global cancers in general are in the downward trend, the incidence of HCC remains an upward trend, increasing by 4.6% from 2005 to 2015.[4] Recent years have witnessed highest age-adjusted incidence rates attributed to chronic hepatitis B virus (HBV) in the male population in Asian, particularly in immigrants from HBV-endemic regions.[5] Fortunately, increasing studies have reported many novel HCC biomarkers and imaging modalities, as well as molecular targets for early detection, prevention, and treatment of this cancer.[6,7]

Long non-coding RNAs (lncRNAs) have emerged as novel therapeutic targets for HCC because of their aberrant expression in disease states and functional cellular roles from the cancer perspective.[8] lncRNAs may participate in various aspects of cellular homeostasis, such as proliferating, growth, and migrating processes or genomic stability.[9] Recent studies have demonstrated lncRNA small nucleolar RNA host gene 1 (SNHG1) as a tumor promoter in human cancers, including breast, cervical, non-small cell lung, and gastric cancers.[10–13] More importantly, SNHG1 has been shown to facilitate the development of HCC and hence exert promise functioning as a therapeutic target for this malignancy.[14,15] However, its downstream mechanism in promoting HCC remains largely undefined.

An intricate interplay exists among various RNA species, consisting of protein-coding mRNAs and non-coding RNAs, including lncRNAs, which are considered as competing endogenous RNAs (ceRNAs) or natural sponges of microRNAs (miRNAs or miRs) by competing for binding to the shared miRNAs.[10] miR-376a is also implicated in diverse types of cancers and can inhibit or promote cancers, depending on the type. For example, miR-376a suppresses proliferation and invasion of non-small-cell lung cancer and osteosarcoma cells.[16] By contrast, miR-376a also promotes the proliferation and metastases of ovarian cancer.[17] It appears that miR-376a suppresses the proliferation and promotes apoptosis in HCC.[18]

Forkhead box protein K1 (FOXK1) is a transcription factor that regulates aerobic glycolysis.[19] FOXK1 has been demonstrated as an oncogene in various cancers through enhancing invasion and metastasis, such as colorectal, prostate, and lung cancers.[20–22] Interestingly, FOXK1 has been revealed to upregulate zinc-finger protein SNAI1

Received 25 August 2020; accepted 1 February 2021;
https://doi.org/10.1016/j.omto.2021.02.002.

Correspondence: Aijin Zhou, Department of Emergency, Linyi People's Hospital, No. 27, Eastern Jiefang Road, Linyi 276000, Shandong Province, P.R. China.
E-mail: zhouaijin5959@163.com

This is an open access article under the CC BY license (http://creativecommons.org/licenses/by/4.0/).

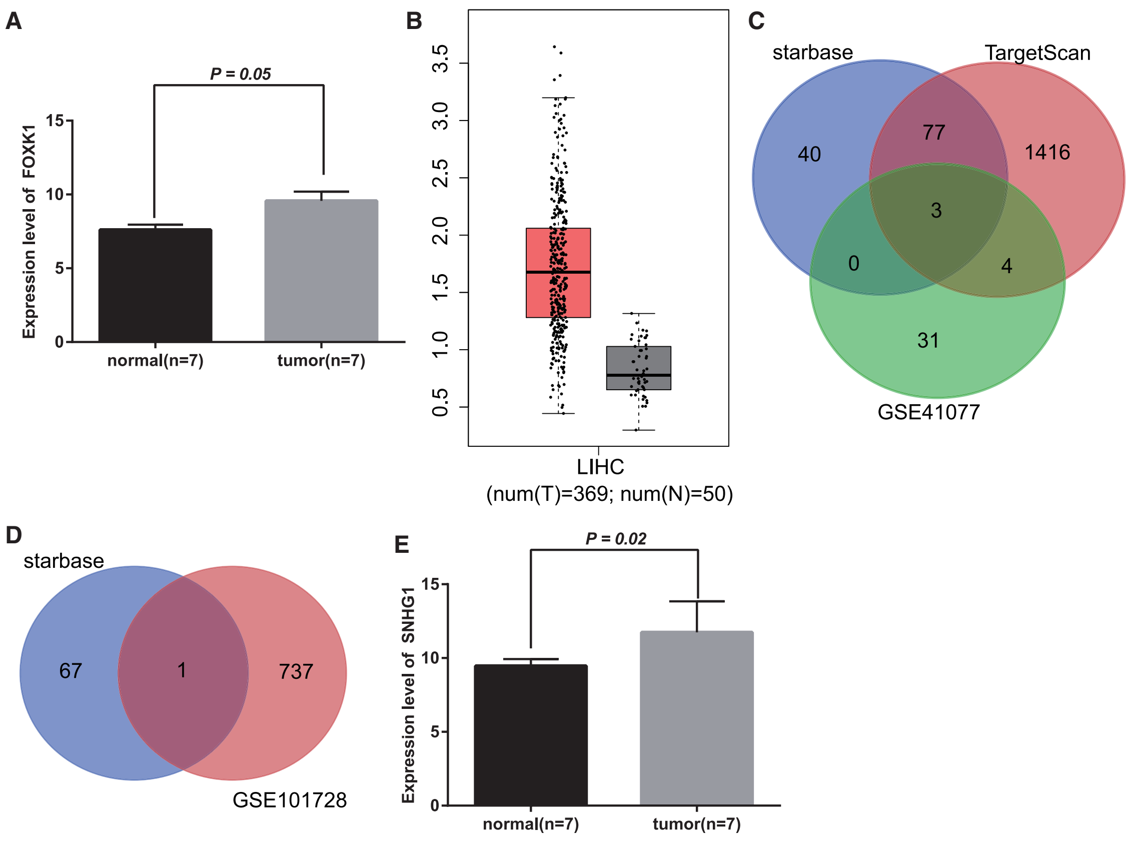

Figure 1. SNHG1 and FOXK1 expressions are increased, whereas miR-376a expression is decreased in HCC

(A) Expression of FOXK1 in normal and tumor samples based on GEO: GSE101728 (n = 7). (B) Expression of FOXK1 in HCC in GEPIA database (http://gepia.cancer-pku.cn/). (C) Upstream miRNAs that could regulate FOXK1 predicted by starBase (http://starbase.sysu.edu.cn/) and TargetScan (http://www.targetscan.org/vert_71/) and miRNAs downregulated in HCC obtained from GEO: GSE41077. (D) lncRNAs that bound to miR-376a predicted by starBase and lncRNAs upregulated in HCC obtained from GEO: GSE101728. (E) Expression of SNHG1 in normal and tumor samples based on GEO: GSE101728 (n = 7).

(Snail).[23] Snail has been shown to be critical to cancer invasiveness and metastasis.[24] Of crucial importance, Snail promotes the metastasis of HCC by mediating epithelial-mesenchymal transition (EMT).[25] In light of the predicted interaction between SNHG1 and miR-376a, as well as between miR-376a and FOXK1, we investigated whether SNHG1, miR-376a, FOXK1, and Snail would be involved in the growth and metastasis of HCC and substantiated their interactions.

RESULTS

SNHG1 and FOXK1 are upregulated and miR-376a is downregulated in HCC

FOXK1 was found to be highly expressed in HCC based on Gene Expression Omnibus (GEO): GSE101728 (Figure 1A), which was consistent with the data obtained from the Gene Expression Profiling Interactive Analysis (GEPIA) database (Figure 1B). Meanwhile, 120 and 1,500 upstream microRNAs (miRNAs) were predicted to target FOXK1 by starBase and TargetScan, respectively. By comparing the aforementioned upstream miRNA candidates obtained from the two databases and miRNAs that were downregulated in HCC based on GEO: GSE41077, miR-199a-5p, miR-376a-3p, and miR-139-5p were found in the intersection (Figure 1C). Through literature review, the interaction between miR-376a and FOXK1 has not been mentioned yet, so we selected the miR-376a as our study object. Furthermore, lncRNA SNHG1 was presumed to have a binding site on miR-376a by starBase and was also significantly upregulated in HCC based on GEO: GSE101728 (Figures 1D and 1E). Hence we speculate that lncRNA SNHG1, miR-376a, and FOXK1 might participate in the biology of HCC because of their dysregulation in HCC and their putative interaction.

High expression of SNHG1 correlates with poor prognosis of patients

By conducting quantitative reverse transcription polymerase chain reaction (qRT-PCR) to quantify RNA levels in clinical HCC and para-cancerous tissues, we confirmed SNHG1 to be markedly increased in cancerous tissues in patients with HCC (Figure 2A). Consistently, SNHG1 was also determined to be highly expressed

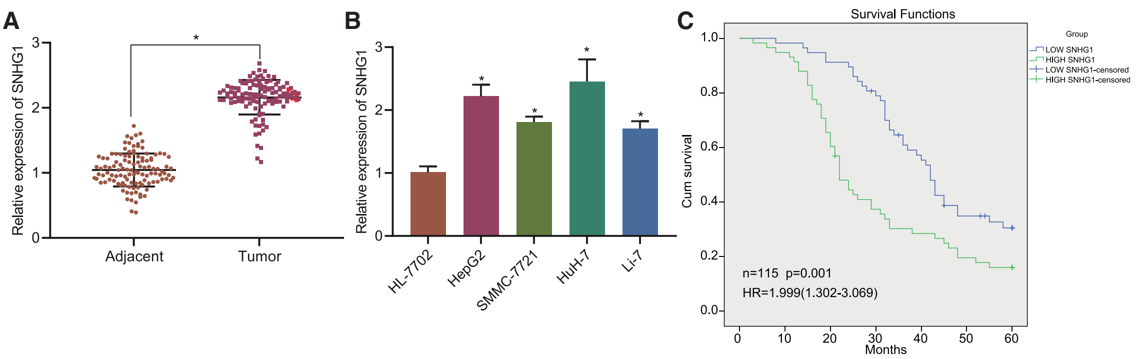

Figure 2. SNHG1 high expression is associated with poor prognosis of patients with HCC

(A) SNHG1 expression in HCC tissues and para-cancerous tissue (n = 115) determined by qRT-PCR. (B) SNHG1 expression in liver cancer cell lines HepG2, SMMC-7721, HuH-7, and Li-7 and normal liver cells HL-7702 determined by qRT-PCR. (C) The relationship between SNHG1 expression and patient survival analyzed by log rank test; *$p < 0.05$ versus para-cancerous tissues or normal liver cells. Data were expressed as mean ± standard deviation. Data between two groups were compared with paired t test. Data among multiple groups were compared with one-way ANOVA with Tukey's post hoc test. Each experiment was repeated three times independently.

in liver cancer cell lines (HepG2, SMMC-7721, HuH-7, and Li-7) as compared with normal liver cells HL-7702 (Figure 2B). Among those liver cancer cell lines, HepG2 and HuH-7 cell lines exhibited relatively higher expression of SNHG1; therefore, HepG2 and HuH-7 cells were selected for subsequent experiments. Moreover, we selected the median value of SNHG1 expression (2.204) as the cutoff value and grouped the 115 patients into the high-expression and low-expression groups. The results showed correlations of high SNHG1 expression with advanced tumor-node-metastasis (TNM) stage, larger tumor diameter, and presence of lymph node metastasis (Table 1), but no correlation with patient age and degree of differentiation. Kaplan-Meier analysis also showed shorter overall survival in patients with higher SNHG1 expression in HCC tissues (Figure 2C). Hence SNHG1 was speculated to potentially affect the progression of HCC.

SNHG1 knockdown inhibits HCC cell proliferation and invasion and promotes apoptosis

For verification purpose of the above speculation, we constructed two short hairpin RNAs (shRNAs) to knock down SNHG1. Both shRNA-1 targeting SNHG1 (sh-SNHG1-1) and sh-SNHG1-2 successfully reduced SNHG1 expression in HuH-7 and HepG2 cells (Figure 3A), among which sh-SNHG1-1 was more effective, so sh-SNHG1-1 was selected for subsequent experiments. Subsequently, the behaviors of HuH-7 cells were evaluated by Cell Counting Kit-8 (CCK-8), flow cytometry, and Transwell assays. SNHG1 knockdown functionally led to suppressed cell viability (Figure 3B), enhanced cell apoptosis (Figure 3C), and inhibited cell invasion and migration (Figure 3D). At molecular levels, migration-associated proteins (matrix metalloproteinase-2 [MMP-2] and MMP-9), apoptosis-associated proteins B cell lymphoma-2 (Bcl-2) and Bcl-2 associated protein X (Bax), and EMT-associated proteins (N-cadherin and E-cadherin) were measured by western blot assay. SNHG1 knockdown reduced the expression of MMP-2, MMP-9, Bcl-2, and N-cadherin while increasing the expression of Bax and E-cadherin (Figure 3E). To prove the silencing effect of sh-SNHG1-1 without off-target effects, we overexpressed SNHG1 when SNHG1 was knocked down, followed by determination on resultant SNHG1 expression and expression of migration and apoptosis-related proteins (Figure S1). Results validated the silencing efficiency of sh-SNHG1-1 without off-target effects. Additionally, expressions of SNHG1 and related proteins affected by sh-SNHG1-2 were also measured. It was found that sh-SNHG1-2 significantly silenced SNHG1 expression and suppressed cell proliferation, migration, and invasion, as well as promoted cell apoptosis. Also, SNHG1 knockdown upregulated migration and invasion-related proteins (MMP-2, MMP-9, and N-cadherin), EMT-related E-cadherin, and apoptosis-related Bax and downregulated Bcl-2 (Figure S2). Taken together, these results demonstrated that SNHG1 downregulation exerted inhibitory effects on HCC proliferation and invasion in contribution to cell apoptosis.

SNHG1 binds to and downregulates miR-376a

The binding region between SNHG1 and miR-376a was shown by starBase (Figure 4A). Based on this binding region, we constructed the wild-type (WT)-SNHG1 and mutant (MUT)-SNHG1 plasmids and conducted dual-luciferase reporter gene assay to verify their binding relationship. miR-376a mimic had no significant effect on luciferase activity of the MUT-SNHG1 but reduced the luciferase activity of WT-SNHG1 in 293T cells (Figure 4B). Furthermore, miR-376a expression was determined to be reduced in HCC tissues (Figure 4C) and HuH-7 and HepG2 cell lines (Figure 4D). RNA-fluorescence *in situ* hybridization (FISH) showed that miR-376a and SNHG1 were mainly expressed in the cytoplasm (Figure 4E). Pearson's correlation analysis displayed a negative correlation between SNHG1 and miR-376a (Figure 4F). Finally, SNHG1 knockdown increased miR-376a expression, while SNHG1 overexpression decreased miR-376 expression in HuH-7 and HepG2 cells (Figure 4G), suggesting that SNHG1 negatively regulated the expression of miR-376a.

Table 1. Correlation analysis between SNHG1 expression and clinicopathological characteristics in liver cancer

Variables	Cases (n = 115)	SNHG1 expression High (n = 58)	SNHG1 expression Low (n = 57)	p value
Age (years)				
≥46	56	30	26	0.577
< 46	59	28	31	
Tumor diameter (cm)				
≥7	59	47	12	<0.001
<7	56	11	45	
Differentiation				
High/moderate	64	30	34	0.455
Low	51	28	23	
Lymph node metastasis				
N0	66	13	53	<0.001
N1/N2	49	45	4	
TNM stage				
Stage I–II	64	11	53	<0.001
Stage III	51	47	4	

χ^2 test was used to test the association between two categorical variables; $p < 0.05$, which was considered as a significant difference. SNHG1, small nucleolar RNA host gene 1; TNM, tumor-node-metastasis.

SNHG1 upregulates FOXK1 expression through binding to miR-376a

Based on the predicted binding region between miR-376a and FOXK1 (Figure 5A), WT-FOXK1-3′ untranslated region (UTR) and MUT-FOXK1-3′ UTR plasmids were designed and generated. Dual-luciferase reporter gene assay was utilized to verify their binding relationship. The results exhibited that miR-376a mimic had no significant effect on the luciferase activity of MUT-FOXK1-3′ UTR while reducing the luciferase activity of WT-FOXK1-3′ UTR in 293T cells (Figure 5B). Additionally, miR-376a knockdown elevated the FOXK1 mRNA expression, while miR-376a overexpression diminished the FOXK1 mRNA expression in HuH-7 and HepG2 cells (Figure 5C). FOXK1 was determined to be upregulated in HCC tissues (Figure 5D) and cell lines (Figure 5E) by qRT-PCR. Pearson's correlation analysis revealed a negative correlation between miR-376a and FOXK1 (Figure 5F) but a positive correlation between SNHG1 and FOXK1 (Figure 5G). The evidence provided by RNA binding protein immunoprecipitation (RIP) exhibited the enrichment of miR-376a, SNHG1, and FOXK1 in the anti-Argonaute 2 (AGO2) group (Figure 5H), suggesting that miR-376a, SNHG1, and FOXK1 could be co-precipitated with AGO2. Hence SNHG1 and FOXK1 formed a complex with AGO2 to competitively bind to miR-376a. Furthermore, SNHG1 overexpression enhanced the binding of miR-376a to SNHG1 but suppressed the binding of miR-376a to FOXK1 in the complex pulled down by AGO2 (Figure 5I). Also, the binding of miR-376a to SNHG1 and FOXK1 was significantly elevated in the complex pulled down by AGO2 relative to immunoglobulin G (IgG). Moreover, SNHG1 knockdown impaired the binding of miR-376a to SNHG1 but potentiated the binding of miR-376a to FOXK1 (Figure 5J). In comparison with IgG, binding between miR-376a and SNHG1 was reduced, while binding between miR-376a and FOXK1 was increased, in the presence of AGO2. Knockdown of SNHG1 alone decreased the FOXK1 expression and increased miR-376a expression, whereas miR-376a loss of function rescued the FOXK1 expression in the presence of sh-SNHG1 in HuH-7 and HepG2 cells (Figures 5K and 5L). Therefore, we concluded that SNHG1 upregulated FOXK1 expression by competitively binding to miR-376a.

miR-376a downregulates Snail expression through targeting FOXK1

After identifying the SNHG1/miR-376a/FOXK1 axis, study focus was subsequently shifted to the FOXK1 downstream Snail. Binding sites between FOXK1 and Snail were predicted by UCSC and JASPAR. Then, JASPAR was used to analyze binding motifs between FOXK1 and Snail as shown in Figure S3A. According to JASPAR, there were three binding sites between FOXK1 and Snail, 251–264, 373–386, and 1841–1854. Binding sites on Snail were mutated (Figure S3B). Snail expression was determined to be highly expressed in HCC tissues (Figure 6A) and cell lines (Figure 6B). Immunohistochemistry consistently showed upregulation of positive FOXK1 and Snail expression in HCC tissues (Figure 6C). Dual-luciferase reporter gene assay was followed for whether FOXK1 could activate Snail expression transcriptionally by binding to the Snail promoter region. Results identified that FOXK1 could bind to the Snail promoter region (Figure 6D). Pearson's correlation analysis concordantly suggested a positive correlation between FOXK1 and Snail (Figure 6E). Through transfection with overexpressed FOXK1 (oe-FOXK1) in HuH-7 and HepG2 cells, FOXK1 expression was elevated and Snail expression was consequently increased (Figure 6F). On the contrary, sh-FOXK1-1 and sh-FOXK1-2 reduced the expression of FOXK1 (Figure 6G), where sh-FOXK1-2 with better efficiency was selected for subsequent experiments. Meanwhile, miR-376a inhibitor efficiently reduced the miR-376a expression and consequently upregulated the FOXK1 and Snail expression, while FOXK1 knockdown diminished the Snail expression in the presence of miR-376a inhibitor (Figures 6H and 6I). These results collectively suggested that miR-376a downregulated Snail expression through binding to FOXK1.

SNHG1 knockdown delays HCC progression *in vitro* through downregulating the miR-376a/FOXK1/Snail axis

SNHG1 knockdown by shRNA reduced the FOXK1 and Snail expression and increased miR-376a expression while Snail expression was restored by co-transfection with plasmids harboring oe-Snail in HuH-7 and HepG2 cells (Figures 7A and 7B). Subsequent CCK8 and Transwell assays revealed that SNHG1 knockdown resulted in suppressed viability (Figure 7C), as well as cell invasion and migration (Figure 7D). However, restoration of Snail normalized these effects. Moreover, SNHG1 knockdown decreased the expression of MMP-2, MMP-9, Bcl-2, and N-cadherin and increased the expression of Bax and E-cadherin, whereas restoration of Snail reversed the changes caused by SNHG1 knockdown (Figure 7E). Taken together, SNHG1

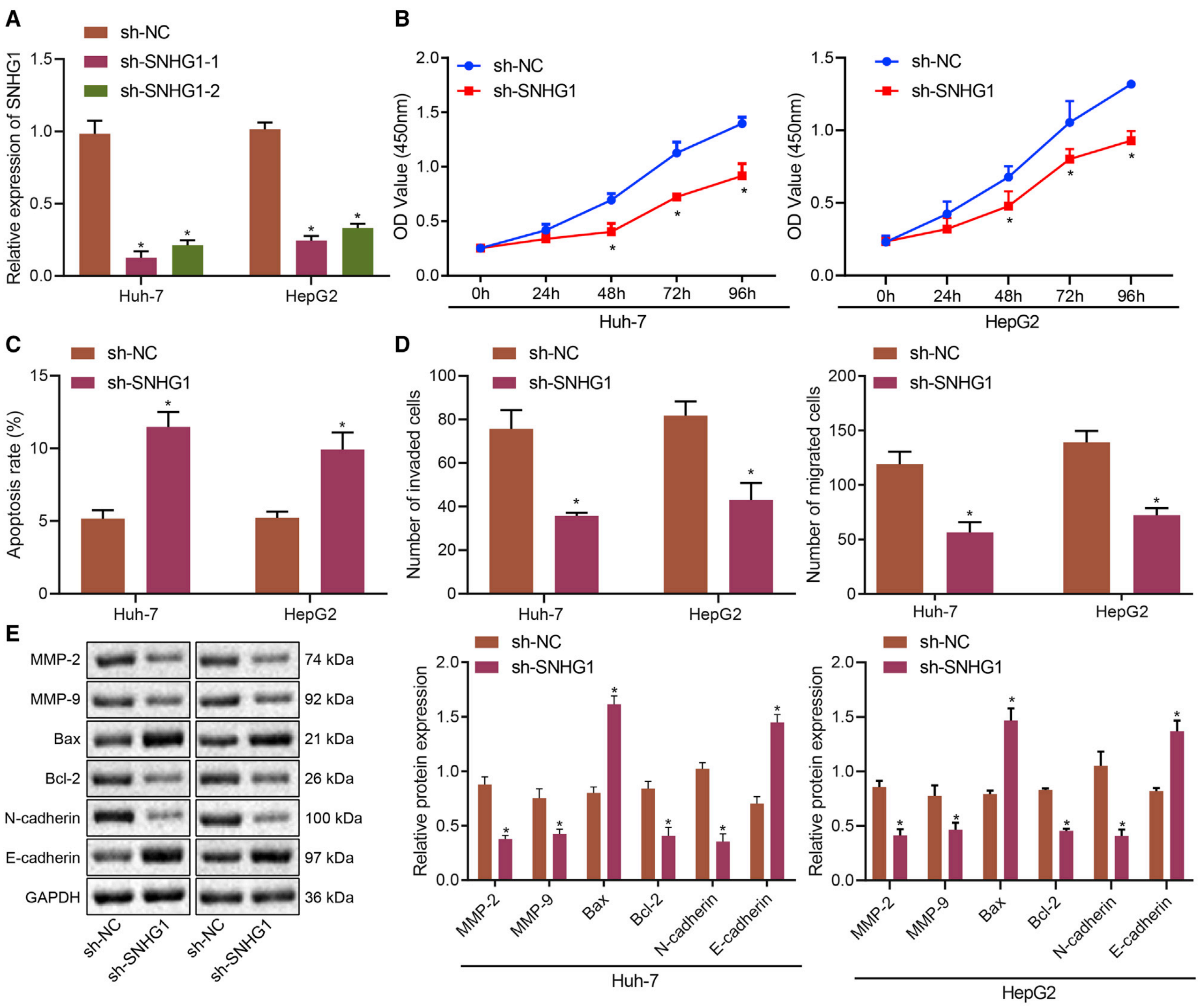

Figure 3. SNHG1 knockdown impedes HCC cell proliferative, migrating, and invasive properties and promotes cell apoptosis

(A) Efficiency of SNHG1 knockdown in HuH-7 cells after transfection with shRNAs. (B) Cell viability after SNHG1 knockdown assessed by CCK8 assay. (C) Cell apoptosis after SNHG1 knockdown assessed by flow cytometry. (D) Cell invasion and migration after SNHG1 knockdown assessed by Transwell assay. (E) Protein expression of MMP-2, MMP-9, Bax, Bcl-2, E-cadherin, and N-cadherin. *$p < 0.05$ versus cells transfected with sh-NC. Data were expressed as mean ± standard deviation. Data between two groups were compared with unpaired t test. Data among multiple groups were compared with one-way ANOVA with Tukey's post hoc test. Data comparison among groups at different time points was performed using repeated-measures ANOVA with Bonferroni post hoc test. Each experiment was repeated three times independently.

knockdown impeded the malignant characteristics of HCC cells by downregulating Snail.

SNHG1 knockdown restrains the tumor growth and metastasis *in vivo* by downregulating Snail

Finally, *in vivo* tumor formation and metastasis were evaluated to show the effect of the SNHG1-mediated miR-376a/FOXK1/Snail axis. SNHG1 knockdown reduced tumor volume and weight (Figure 8A) and lung metastasis (Figures 8B and 8C), while the restoration of Snail reversed those reductions. SNHG1 knockdown decreased MMP-2 but increased E-cadherin expression, whereas Snail restoration normalized those changes caused by SNHG1 knockdown (Figure 8D). Hence SNHG1 knockdown slows down the growth and metastasis of HCC by downregulating Snail.

DISCUSSION

In the current study, we attempted to better understand the pathological mechanisms in HCC, therefore seeking more effective and noninvasive treatments. There are a few important findings in this study. SNHG1 was determined to be an upregulated lncRNA in HCC, and

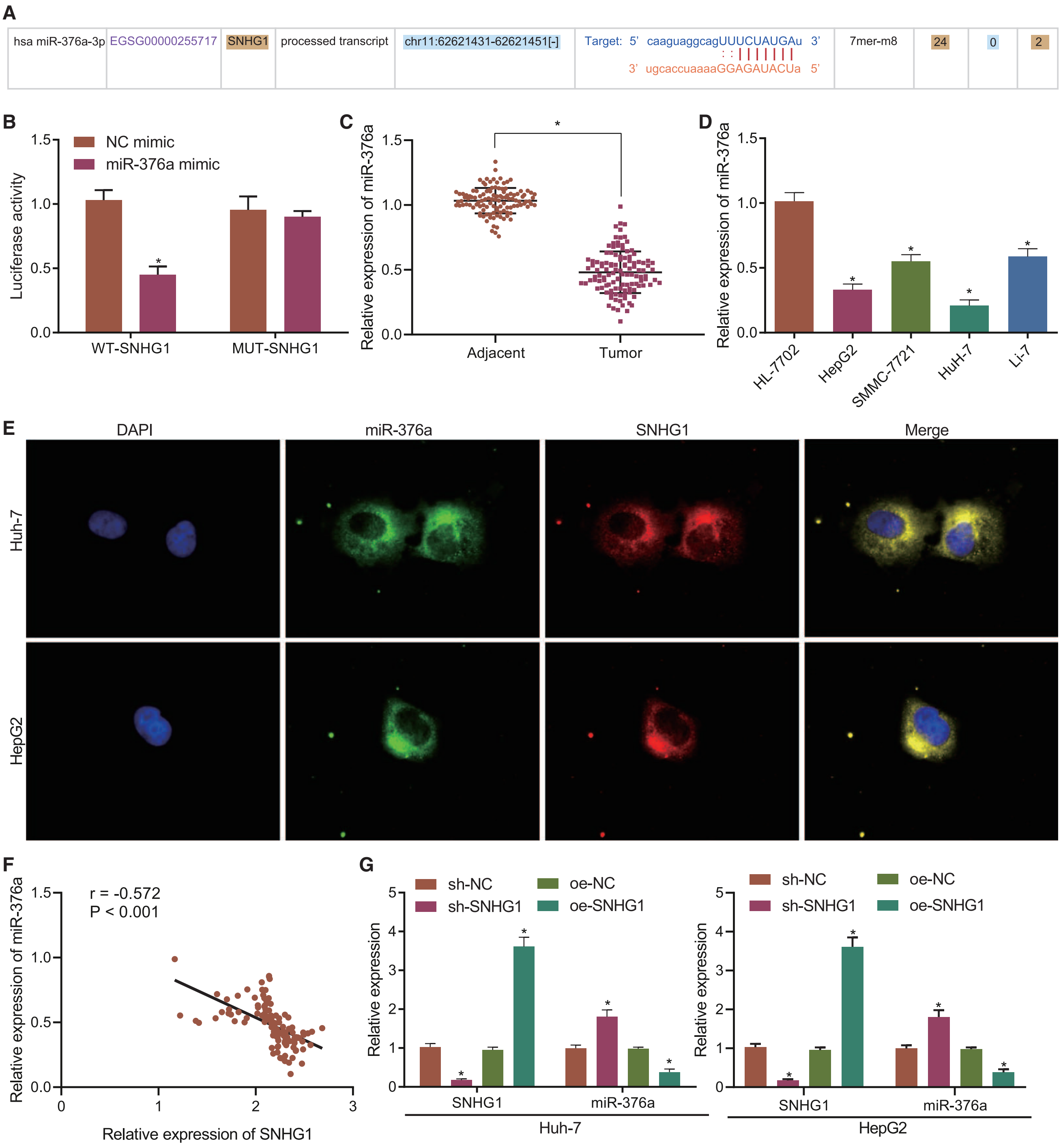

Figure 4. SNHG1 binds to and downregulates miR-376a

(A) The binding of SNHG1 to miR-376a predicted by starBase. (B) Binding relationship between SNHG1 and miR-376a determined by dual-luciferase reporter gene assay. (C) miR-376a expression in HCC and para-cancerous tissues (n = 115) determined by qRT-PCR. (D) miR-376a expression in liver cancer cell lines HepG2, SMMC-7721, HuH-7, and Li-7 and normal liver cells HL-7702 determined by qRT-PCR. (E) miR-376a and SNHG1 localization determined by RNA-FISH (original magnification 400×). (F) Pearson's correlation analysis of SNHG1 and miR-376a. (G) miR-376a expression after SNHG1 overexpression or knockdown determined by qRT-PCR. *$p < 0.05$ versus para-cancerous tissues, normal liver cells, or cells transfected with sh-NC or oe-NC. Data were expressed as mean ± standard deviation. Data between two groups were compared with unpaired t test. Data among multiple groups were compared with one-way ANOVA with Tukey's post hoc test. Each experiment was repeated three times independently.

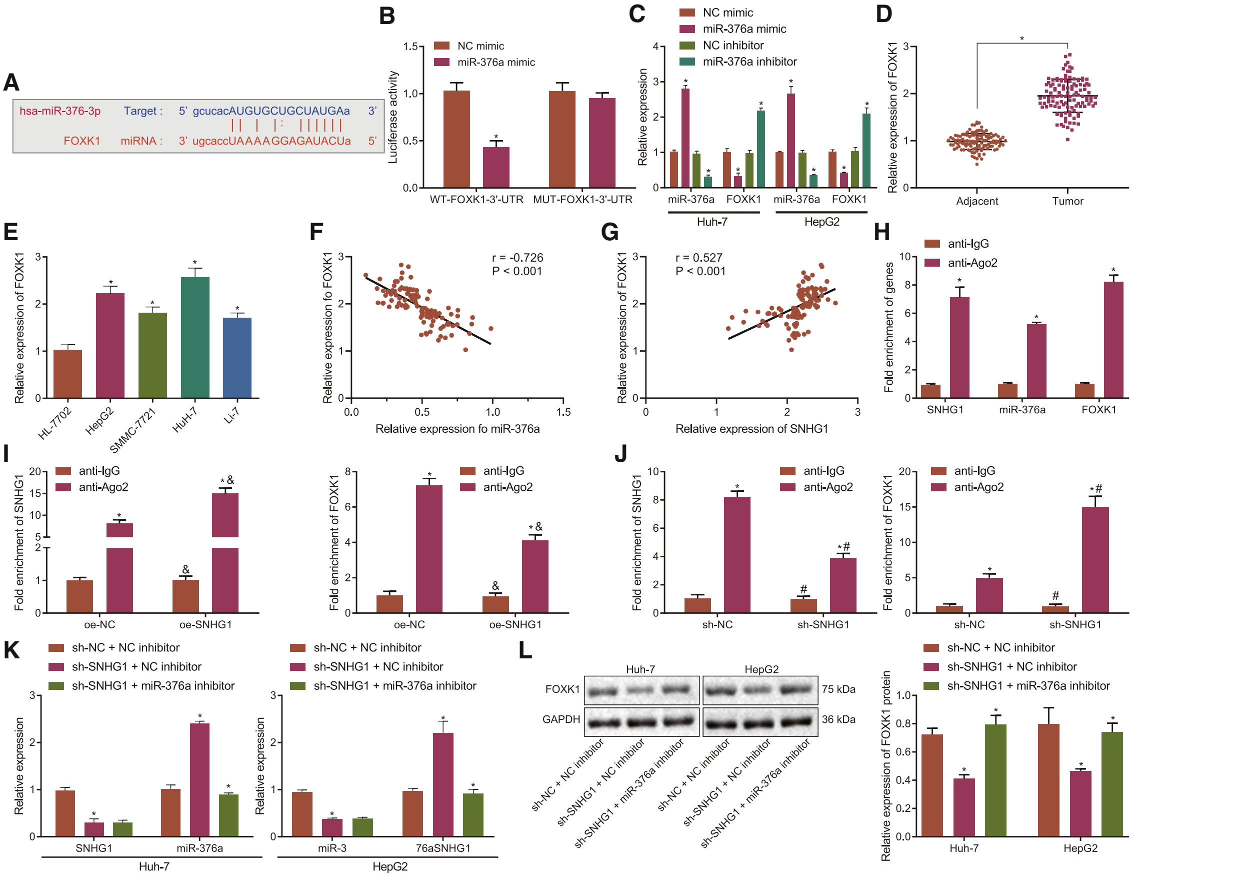

Figure 5. SNHG1 positively regulates FOXK1 expression through binding to miR-376a

(A) The binding region between miR-376a and FOXK1. (B) Binding relationship between miR-376a and FOXK1 determined by dual-luciferase reporter gene assay. (C) miR-376a and FOXK1 expression after transfection with miR-376a mimic or inhibitor determined by qRT-PCR. (D) FOXK1 mRNA expression in HCC and para-cancerous tissues (n = 115) determined by qRT-PCR. (E) FOXK1 mRNA expression in liver cancer cell lines HepG2, SMMC-7721, HuH-7, and Li-7 and normal liver cells HL-7702 determined by qRT-PCR. (F) Pearson's correlation analysis between miR-376a and FOXK1 in cancerous tissues. (G) Pearson's correlation analysis between SNHG1 and FOXK1 in cancerous tissues. (H) Enrichment of miR-376a, SNHG1, and FOXK1 co-precipitated with AGO2 determined by RIP. (I) Binding relationship between miR-376a and SNHG1 or FOXK1 after SNHG1 overexpression determined by RIP. (J) Binding relationship between miR-376a and SNHG1 or FOXK1 after SNHG1 knockdown determined by RIP; *$p < 0.05$ versus anti-IgG, &$p < 0.05$ versus cells transfected with oe-NC, #$p < 0.05$ versus cells transfected with sh-NC. (K) SNHG1 and miR-376a expression after transfection with sh-SNHG1 or in combination with miR-376a inhibitor determined by qRT-PCR. (L) FOXK1 protein expression after transfection with sh-SNHG1 or in combination with miR-376a inhibitor measured by western blot assay; *$p < 0.05$ versus para-cancerous tissues, normal liver cells, anti-IgG, or cells transfected with NC mimic, NC inhibitor, sh-NC, or oe-NC. Data were expressed as mean ± standard deviation. Data between two groups were compared with paired or unpaired t test. Data among multiple groups were compared with one-way ANOVA with Tukey's post hoc test. Each experiment was repeated three times independently.

its knockdown delayed the cancer progression *in vitro* and tumor metastasis *in vivo*. Additionally, SNHG1 could competitively bind to miR-376a, leading to increased FOXK1 and Snail expression, whereby promoting HCC progression.

We found that SNHG1 expression was increased in HCC tissues and cell lines. In addition, its high expression correlated with poor outcome of patients with HCC and advanced tumor stage, which was consistent with the findings reported by Zhang and his team,[15] suggesting a prognostic value of SNHG1. SNHG1 was documented to be an upregulated lncRNA in HCC and had a value of serving as a noninvasive biomarker.[26] Also, plasma SNHG1 holds potential as a sensitive and reliable diagnostic marker of HCC because of its close correlation with tumor size and TNM stage.[27] Consistently, ectopic SNHG4 expression shares an independent association with shorter overall survival in HCC.[28] SNHG1 knockdown was demonstrated in the present study to suppress the growth, migrating, and invading potentials while accelerating apoptosis of HCC cells. Our study also determined the tumor-suppressing function of SNHG1 knockdown at molecular levels, corresponding to reductions in MMP-2, MMP-9, Bcl-2, and N-cadherin and elevations in Bax and E-cadherin. Hence SNHG1 might be a tumor promoter and pro-metastatic lncRNA. The tumor-promotive role of SNHG1 in HCC has also been evidenced by several recent research studies.[29–32] These studies also demonstrated that PDCD4,

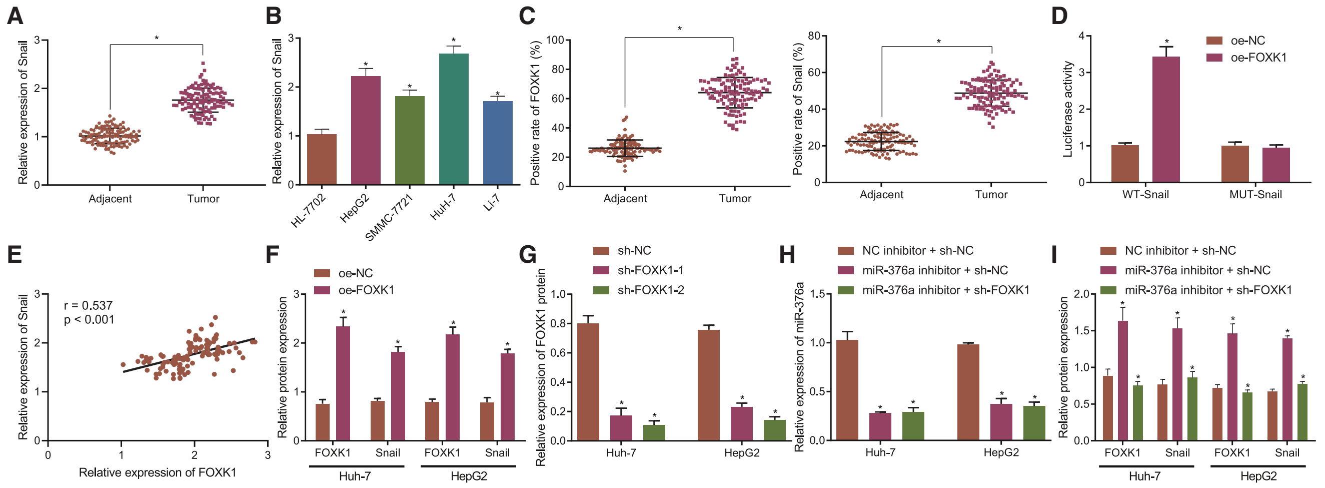

Figure 6. miR-376a negatively regulates Snail expression through downregulation of FOXK1

(A) Snail mRNA expression in HCC and para-cancerous tissues (n = 115) determined by qRT-PCR. (B) Snail mRNA expression in liver cancer cell lines HepG2, SMMC-7721, HuH-7, and Li-7 and normal liver cells HL-7702 determined by qRT-PCR. (C) FOXK1 and Snail expressions in HCC and para-cancerous tissues determined by immunohistochemistry. (D) Binding relationship between FOX1 and Snail determined by dual-luciferase reporter gene assay. (E) Pearson's correlation analysis between FOXK1 and Snail expression. (F) FOXK1 and Snail protein expression after transfection with oe-FOXK1 measured by western blot assay. (G) Efficiency of FOXK1 knockdown. (H) miR-376a expression after co-transfection with miR-376a inhibitor and sh-FOXK1/sh-NC determined by qRT-PCR. (I) FOXK1 and Snail expression after co-transfection with miR-376a inhibitor and sh-FOXK1/sh-NC measured by western blot assay. *$p < 0.05$ versus para-cancerous tissues, normal liver cells, anti-IgG, or cells transfected with sh-NC or oe-NC, or co-transfected with NC inhibitor and sh-NC. Data were expressed as mean ± standard deviation. Data between two groups were compared with unpaired t test. Data among multiple groups were compared with one-way ANOVA with Tukey's post hoc test. Each experiment was repeated three times independently.

Akt pathway, miR-21, and miR-195 may be downstream signaling molecules of SNHG1-mediated promotion of HCC progression. A recent identification of the ceRNA network in HCC suggested that SNHG1 potentially interacted with miRNAs and mRNAs to regulate protein phosphorylation or cell-cycle pathways in HCC,[33] which was also shown by a more recent study.[34] In our study, SNHG1 was predicted to bind to miR-376a, and their binding relationship was identified by dual-luciferase reporter gene and RIP assays.

Another important finding of the study was that SNHG1 bound to miR-376a and suppressed the expression of miR-376a. miR-376a was determined to be downregulated in HCC tissues versus non-tumor tissues.[35] This was largely coincident with our findings. An earlier study demonstrated that miR-376a suppressed the proliferation and promotes apoptosis of HCC.[18] Our results agree with this study and suggested that downregulation of miR-376a might underlie the tumor-promotive action of SNHG1. Moreover, decreased expression of miR-376a led to increased FOXK1 expression as shown by our study. miR-376a was confirmed to target FOXK1, while FOXK1 was identified to bind to Snail through luciferase assay. FOXK1 has been shown to promote other types of cancers, such as prostate, lung, and colorectal cancers.[20–22] In addition to those cancers, FOXK1 silencing suppressed the proliferative, migrative, and invasive properties of HCC cells.[36] More recent research reported the prognostic value of FOXK1 in HCC and its inhibitory function in the sphere-forming ability of HCC stem cells.[37] Furthermore, FOXK1 knockdown repressed liver cancer cell viability through mediating glycolysis, which was achieved by inhibition of the Akt/mammalian target of rapamycin pathway.[38] Furthermore, our results on Snail are also comparable with a previous study showing that Snail is critical to aggravate cancer invasiveness in liver cancer.[25] Previous studies showed that Snail overexpression could induce EMT in liver cancer, contributing to the liver stem cells transforming to liver cancer stem cells.[39,40] Our study evidenced both *in vitro* and *in vivo* that SNHG1 knockdown impeded cancer progression and tumor metastasis through downregulation of Snail. Of note, FOXK1 has been indicated to facilitate gastric cancer cell growth, EMT, invasion, and metastasis by transcriptionally activating *c-jun*.[41] Additionally, action of FOXK1 in HCC has been reported to depend on the Akt/mTOR pathway as a downstream target of miR-329-3p.[42] Further studies are required to determine the exact regulatory mechanism of FOXK1 in HCC and identify its working partners.

In conclusion, we demonstrated that SNHG1 facilitated HCC development by inhibiting miR-376a and activating FOXK1/Snail (Figure 9). These signaling molecules may be potential new therapeutic targets for the treatment of HCC. However, results from this study should be confirmed in other animal model and cell lines of HCC to better mimic the heterogeneity of the human disease.

MATERIALS AND METHODS

Study subjects

Written informed consents were obtained from all patients, and our experimental protocols were approved by the local ethics committee.

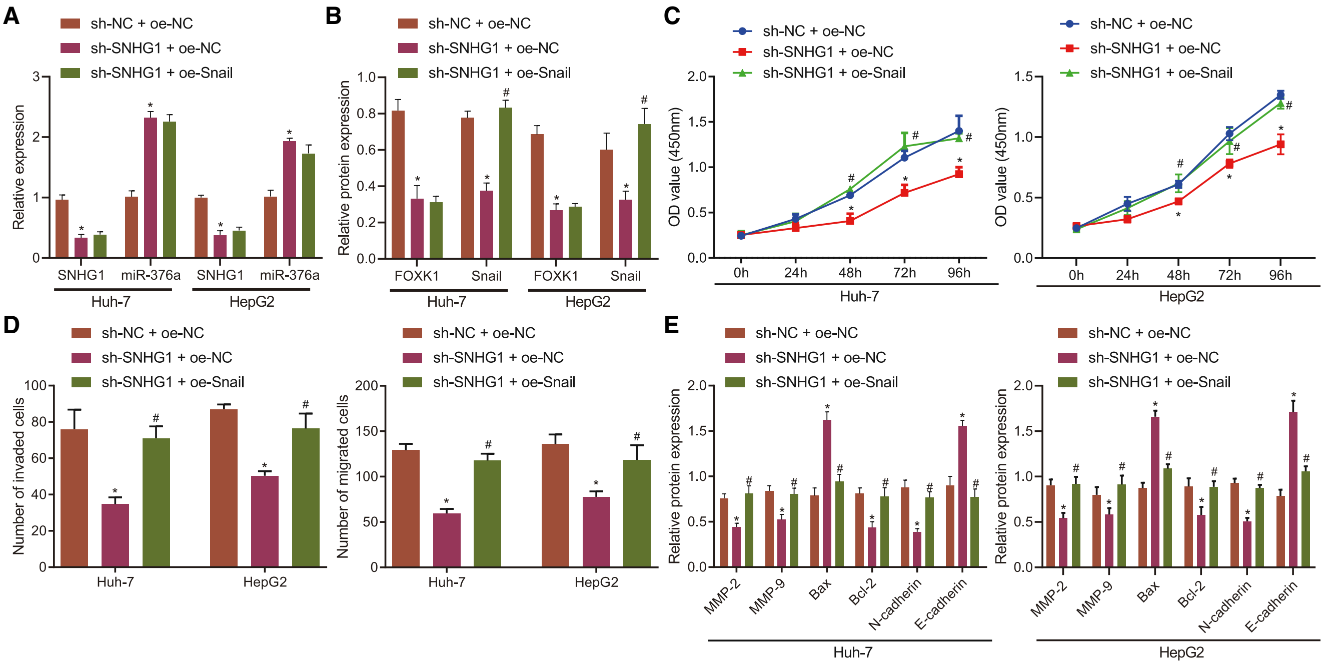

Figure 7. SNHG1 accelerates HCC cell invasiveness and migration through upregulating Snail

(A) SNHG1 and miR-376a expression determined by qRT-PCR. (B) Snail and FOXK1 protein expression measured by western blot assay. (C) Cell viability assessed by CCK8 assay. (D) Cell invasion and migration determined by Transwell assay. (E) Expression of MMP-2, MMP-9, Bax, Bcl-2, E-cadherin, and N-cadherin proteins measured by western blot assay; *$p < 0.05$ versus cells co-transfected with sh-NC and oe-NC; #$p < 0.05$ versus cells co-transfected with sh-SNHG1 and oe-NC. Data were expressed as mean ± standard deviation. Data among multiple groups were compared with one-way ANOVA with Tukey's post hoc test. Data comparison among groups at different time points was performed using repeated-measures ANOVA and Bonferroni post hoc test. Each experiment was repeated three times independently.

All *in vivo* experimental protocols were approved by the ethics committee and the review board of Linyi People's Hospital, and the experiments were conducted in accordance with national guidelines. The use of human tissue samples in this project was approved by the Institutional Review Board of Linyi People's Hospital, and written informed consent was obtained from all patients. The study methodologies conformed to the standards set by the Declaration of Helsinki.

In silico analysis

HCC-related datasets GEO: GSE101728 and GSE41077 were obtained from the GEO database (https://www.ncbi.nlm.nih.gov/geo/). Differentially expressed genes/lncRNAs in GEO: GSE101728 were defined at |log fold change (FC)| >0.75 and p <0.05 using R "limma" package. Differentially expressed miRNAs in GEO: GSE41077 were defined at |logFC| >1 and p <0.01 by GEO platform analysis tool GEO2R.

Patient enrollment

One hundred fifteen patients (aged 37–56 years, an average age of 46.65 ± 5.35 years) who were clinically and pathologically diagnosed with HCC in Linyi People's Hospital from November 2014 to June 2016, including 69 males and 46 females, were randomly selected. The patient cohort was classified on the basis of tumor size and TNM stage in line with American Joint Committee on Cancer tumor staging system (seventh edition). None of the enrolled patients received treatment for HCC in the last 3 months. The complete follow-up data for at least 5 years were collected. The patients who received other therapies, such as radiotherapy, chemotherapy, or targeted therapy, and who were not diagnosed to be HCC after surgical operation were excluded. The cancerous tissues and para-cancerous tissues (adjacent to cancerous tissues) were collected from the enrolled patients with HCC.

Cell culture and transfection

Normal human liver cell line HL-7702 cells (3131C0001000200006; China Center for Type Culture Collection) were cultured in Roswell Park Memorial Institute (RPMI) 1640 medium (12633012; HaoRan Bio, Shanghai, P.R. China) containing 10% fetal bovine serum (FBS). Human liver cancer cell line HepG2 cells (3111C0001CCC000035; China Center for Type Culture Collection) were cultured in standard medium. SMMC-7721 cells (3111C0001CCC000087; China Center for Type Culture Collection) were cultured with 20% FBS-supplemented RPMI 1640 medium (12633012; HaoRan Bio). HuH-7 cells (3111C0001CCC000679; China Center for Type Culture Collection) were cultured in basal culture medium. Li-7 cells (3111C0001CCC000678; China Center for Type Culture Collection) were cultured in RPMI 1640 medium containing 20% FBS. All cells were

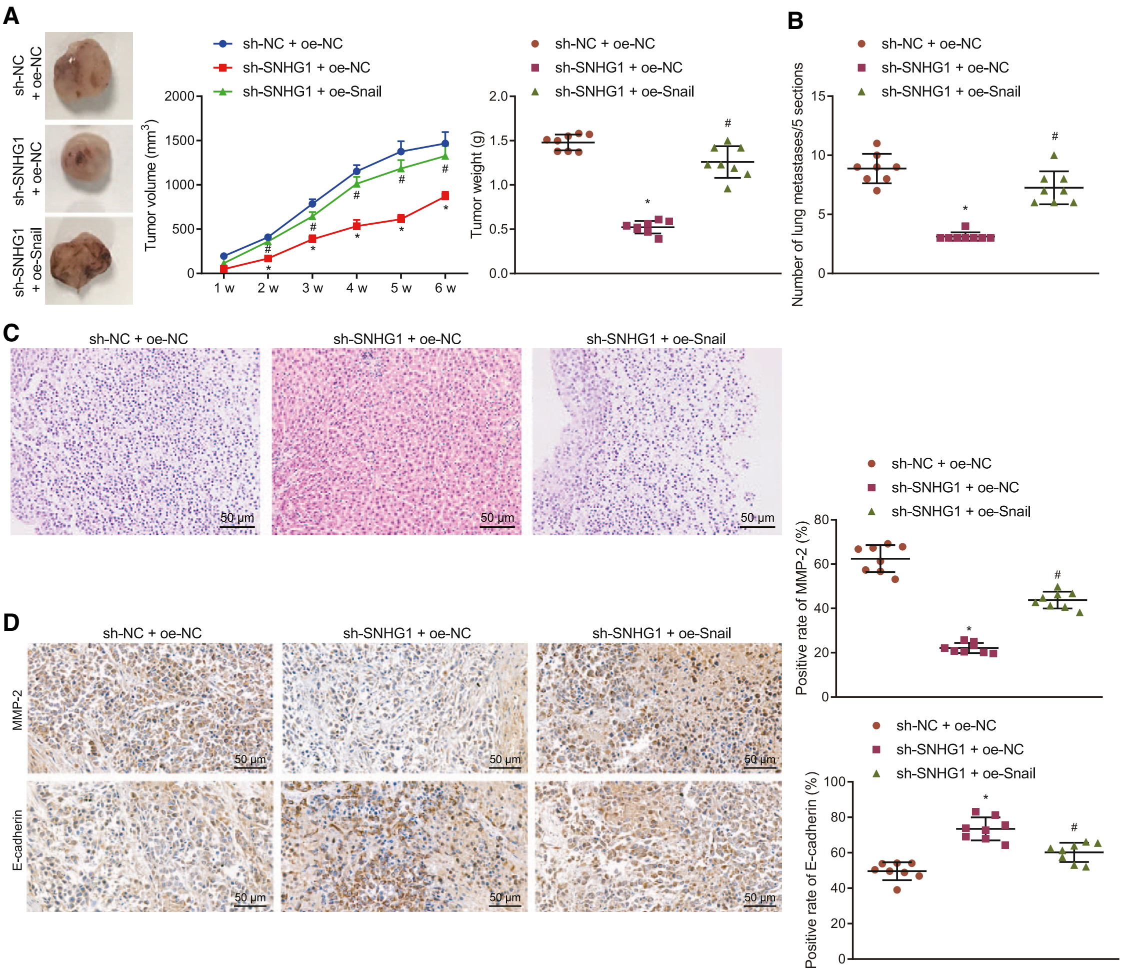

Figure 8. SNHG1 knockdown inhibits the tumor growth and metastasis by downregulating Snail

(A) Representative images, volume, and weight of tumors formed in nude mice. (B) Statistics of lung metastasis nodes in nude mice. (C) The metastasis nodes shown by H&E staining (original magnification 200×). (D) Invasion and migration-related proteins (MMP-2 and E-cadherin) detected by immunohistochemistry (original magnification 400×); #$p < 0.05$ versus nude mice experienced inoculation of the cells co-treated with sh-NC and oe-NC. Data were expressed as mean ± standard deviation. Data among multiple groups were compared with one-way ANOVA with Tukey's post hoc test. Data comparison among groups at different time points was performed using repeated-measures ANOVA and Bonferroni post hoc test. Each experiment was repeated three times independently.

cultured in a 5% CO_2 incubator at 37°C and collected at log growth phase.

Transfection was performed using Lipofectamine 2000 kit (Invitrogen, Carlsbad, CA, USA). The plasmids employed in this study contained sh-negative control (NC), sh-SNHG1, sh-FOXK1, oe-NC, oe-Snail, NC inhibitor, and miR-376a inhibitor. In brief, 4 μg of target plasmid and 10 μL of Lipofectamine 2000 were separately diluted with improved Minimal Essential Medium (250 μL; GIBCO, Waltham, MA, USA). The mixture of two dilutions was added into cells. After incubation for 4 h, serum-free medium was discarded and replaced with serum-containing medium. Following another 4-h incubation, the cells were treated with G418 (400 μg/mL). The medium was renewed every 3 days, and concentration of G418 was reduced for screening until stable cell lines were obtained.

Dual-luciferase reporter gene assay

Synthesized FOXK1-3′ UTR and SNHG1 gene fragments were introduced into pMIR-reporter (Huayueyang Biotechnology, Beijing, P.R. China) using endonuclease sites SpeI and Hind III to design WT FOXK1 and SNHG1 plasmids. The MUT FOXK1 and SNHG1 plasmids were generated with the binding sites mutated so that sequences containing mutated sites were cleaved by restriction enzymes and the target fragment was inserted into the pMIR reporter plasmid using T4 DNA ligase. Sequenced luciferase reporter plasmids WT and MUT were co-transfected with miR-376a mimic into HEK293T cells (Institutes for Biological Sciences, Chinese Academy of Sciences, Shanghai, P.R. China). Forty-eight hours after transfection, cells were collected and lysed. Luciferase activity was detected using a luciferase detection kit (K801-200; Biovision) and a Glomax20/20 luminometer (Promega, Madison, WI, USA).

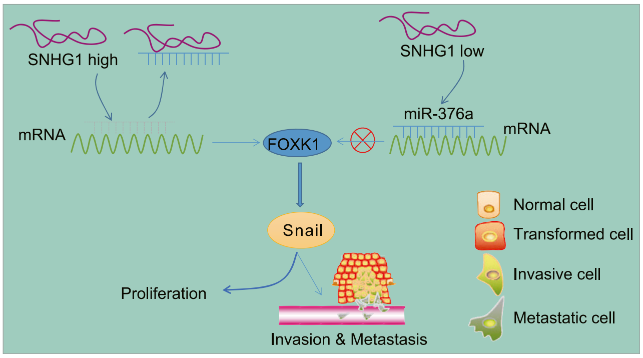

Figure 9. Schematic diagram showing potential mechanism of the SNHG1/miR-376a/FOXK1/Snail axis in HCC growth and metastasis

SNHG1 competitively binds to miR-376a and inhibits the expression of miR-376a, thereby promoting FOXK1 and Snail expression, leading to the promotion of tumor growth and metastasis in HCC.

Based on prediction results available at http://jaspardev.genereg.net/, FOXK1 could bind to the Snail promoter region of 251–264 sites (5′-gaagtaaacagata-3′), 373–386 sites (5′-tccgtaaacactgg-3′), and 1,841–1,854 sites (5′-tccgtaaacactgg-3′). WT and MUT Snail promoter regions were constructed and inserted into pGL3-basic vector (Promega). HEK293 cells were seeded in 24-well plates at 3×10^4 cells/well. WT-Snail and MUT-Snail were co-transfected with oe-NC and oe-FOXK1, respectively, into HEK293T cells. Cells were collected and lysed 48 h after transfection. Luciferase activity was determined by luciferase detection kit (K801-200; Biovision) and a Glomax20/20 luminometer (Promega).

FISH

Subcellular localization of SNHG1 and miR-376a in HCC cells was determined by FISH using Ribo lncRNA FISH Probe Mix (Red) (Ribo Biotechnology, Guangzhou, P.R. China). Cells were seeded at 6×10^4 cells/well in a 24-well culture plate. When cell confluence was about 80%, cells were washed with phosphate-buffered solution (PBS), and 1 mL of 4% paraformaldehyde was added. Cells were fixed at room temperature and permeated with 0.2% TX-100 at ambient temperature for 20 min. Cells were treated with Proteinase K (2 μg/mL), glycine, and acetylphthalating reagent. Pre-hybridization solution (250 μL) was added and incubated at 42°C for 1 h. Hybridization solution containing probe (250 μL, 300 ng/mL) was added and hybridized overnight at 42°C. After being washed three times with PBS buffer containing 0.1% Tween 20 (PBST), 4′-6-diamidino-2-phenylindole (1:800; Thermo Fisher Scientific, Waltham, MA, USA) was added to stain nucleus for 5 min. Cells were washed three times with PBST for 3 min each. Cells were mounted with an anti-fluorescence quencher. Five randomly selected fields were observed under a fluorescence microscope (Olympus, Tokyo, Japan).

shRNA screening

The sh-SNHG1 and sh-FOXK1 sequences were designed based on the GenBank database. BLAST was utilized to exclude shRNAs that specifically inhibit other gene fragments. Two optimal sequences were selected (Table 2) and constructed into the PshRNA-neo plasmid. The generated plasmids were named sh-SNHG1-1, sh-SNHG1-2, sh-FOXK1-1, and sh-FOXK1-2. After transfection into the primary cultured HOSEpiC cells, qPCR was utilized to determine the expression of FOXK1 and SNHG1 in cells in order to select effective shRNA sequences for subsequent experiments.

Table 2. Sequences of shRNAs

shRNAs	Sequences
sh-SNHG1-1	5′-CCTTAAAGTGTTAGCAGACACAGAT-3′
sh-SNHG1-2	5′-GATTAAGACACTGGGAGCCAATGAA-3′
sh-FOXK1-1	5′-TGAACTTCTCCACGATGCACCTATG-3′
sh-FOXK1-2	5′-AACTTCTCCACGATGCACCTATGTT-3′
Negative control	5′-TTCTCCGAACGTGTCACGTTT-3′

sh, short hairpin; shRNA, short hairpin RNA; SNHG1, small nucleolar RNA host gene 1.

RIP

Cells were lysed with an equal volume of Radio-Immunoprecipitation Assay lysis buffer (P0013B; Beyotime, Shanghai, P.R. China) in an ice bath for 5 min. Cells were centrifuged at 14,000 rpm at 4°C for 10 min to obtain the supernatant. Part of the supernatant was preserved as input. The remaining portion was incubated with antibody to AGO2 (ab32381, 1:50; Abcam, Cambridge, UK) or IgG (1:100, ab109489; Abcam) at ambient temperature for 30 min. Magnetic beads (50 μL) were rinsed and resuspended in 100 μL RIP wash buffer. Antibody (5 μg) was added and incubated for binding. The magnetic bead-antibody complex was washed and resuspended in 900 μL RIP wash buffer. Cell lysate (100 μL) was added and incubated overnight at 4°C. Samples were placed on a magnetic holder to collect magnetic bead-protein complex. Samples and input were digested with Proteinase K to extract RNA for subsequent PCR analysis for SNHG1 and FOXK1.

CCK8 assay

Cells were seeded into a 96-well plate at 2×10^3 cells/well. Only medium without cells was set for blank. After cell transfection for 24, 48, 72, and 96 h, 10 μL of CCK8 solution was added to each well and incubated for 4 h at 37°C. Absorbance at 450 nm was measured using a microplate reader (Bio-Rad, Hercules, CA, USA).

Flow cytometry

On the second day after transfection, cells were detached with 0.25% trypsin, which was terminated by RPMI-1640 medium containing 10% FBS. Cells were centrifuged at 1,000 rpm for 5 min. Supernatant was discarded. Cells were fixed by pre-cooled 70% ethanol at 4°C. Cell concentration was adjusted to 1×10^6 cells/mL. Annexin V-fluorescein isothiocyanate/propidium iodide (FITC/PI) (10 mL, 556547; Surej Biotechnology, Shanghai, P.R. China) was then added, and cells were stained in a 4°C refrigerator for 15–30 min. FITC was detected at

Table 3. Primer sequences for qRT-PCR

Primer	Sequences
miR-376a	forward: 5′-GTGCAGGGTCCGAGGT-3′
	reverse: 5′-ATCATAGAGGAAAATCCACG-3′
SNHG1	forward: 5′-CCGCTCGAGATTTAGGTGACACTATAGAAGTTCT CATTTTTCTACTGCTCG-3′
	reverse: 5′-ATAGTTTAGCGGCCGCTTTTTTTTTTTTTTTTTAT GTAATCAATCATTTTAT-3′
U6	forward: 5′-GCTTCGGCAGCACATATACTAAAAT-3′
	reverse: 5′-CGCTTCACGAATTTGCGTGTCAT-3′
β-actin	forward: 5′-AAGAGCTACGAGCTGCCTGA-3′
	reverse: 5′-GGCAGTGATCTCCTTCTGCA-3′

miR, microRNA; qRT-PCR, quantitative reverse transcription polymerase chain reaction; SNHG1, small nucleolar RNA host gene 1.

530 nm, and PI was detected at 575 nm on a flow cytometer (type XL; Coulter, Brea, CA, USA) with an excitation wavelength of 480 nm.

Transwell assay

The cell invasion was assessed by Transwell assay using Matrigel-coated Transwell chamber (MAMIC8S10; Millipore Corporation, USA). In detail, cells 1×10^5 cultured in serum-free medium were seeded into Matrigel-coated Transwell chamber. DMEM containing 10% FBS (500 μL) was added to the lower chamber. After 24 h of incubation at 37°C and 5% CO_2, the non-invaded cells were removed. Transwell chamber was washed twice with PBS, fixed with 100% methanol for 30 min, and stained with 0.5% crystal violet (C3886; Sigma-Aldrich, St. Louis, MO, USA) for 20 min. The invaded cells were observed under an inverted fluorescence microscope (TE2000; Nikon, Tokyo, Japan). Five fields were randomly observed and imaged.

The cell migration was also assessed by Transwell assay using Transwell chamber without Matrigel. The serum-free medium containing 1×10^6 cells/mL was added to the upper chamber. Cells were incubated at 37°C for 20–24 h. The following procedures were the same as the above-described Transwell assay for cell invasion. After drying, cells in five random fields were counted under the inverted microscope.

qRT-PCR

Total RNA was extracted using TRIzol kit (15596026; Invitrogen). RNA was reversely transcribed to cDNA using a reverse transcription kit (RR047A; Takara, Kyoto, Japan). RNA reaction system contained 20 μL, and reaction conditions consisted of 37°C for 15 min and 85°C for 5 s. TaqMan MicroRNA Assay and TaqMan Universal PCR Master Mix were employed for real-time qPCR of miRNA. The PCR conditions included 95°C for 2 min, followed by 45 cycles at 95°C for 15 s and 60°C for 45 s. SYBR Premix EX Taq Kit (RR420A; Takara) was utilized for qPCR of mRNA, which was performed in an ABI7500 system (ABI, Foster City, CA, USA). Samples were tested in triplicates. Primer sequences are shown in Table 3 (Sangon, Shanghai, P.R. China). The expression of target genes relative to β-actin and miRNA relative to U6 was calculated by the $2^{-\Delta\Delta Ct}$ method.

Western blot assay

Total protein was extracted from cells. Protein concentration was measured using a bicinchoninic acid kit (Thermo). Protein (30 μg) was subjected to polyacrylamide gel electrophoresis at 80 V for 35 min and 120 V for 45 min. After the electrophoresis was completed, proteins were transferred to a polyvinylidene fluoride membrane. Membrane was blocked by 5% skim milk at ambient temperature for 1 h. Membranes were incubated with rabbit anti-FOXK1 (1:1,000, ab18196; Abcam), rabbit anti-Snail (1:1,000, ab180714; Abcam), rabbit anti-MMP-2 (1:1,000, ab97779; Abcam), rabbit anti-MMP-9 (1:1,000, ab73734; Abcam), rabbit anti-Bcl-2 (1:2,000, ab182858; Abcam), rabbit anti-Bax (1:2,000, ab32503; Abcam), rabbit anti-E-cadherin (1:500,000, ab76319; Abcam), rabbit anti-N-cadherin (1:2,000, ab18203; Abcam), and rabbit anti-glyceraldehyde-3-phosphate dehydrogenase (GAPDH; 1:2,500, ab9485; Abcam) at 4°C overnight. Membranes were incubated with horseradish peroxidase-labeled goat anti-rabbit IgG antibody (1:10,000, ab6721; Abcam) for 1 h at ambient temperature after PBST washing. Membranes were washed three times with PBST buffer for 10 min each. After development with an optical luminometer (GE, Boston, MA, USA), gray intensity of protein bands was measured by Image ProPlus 6.0 (Media Cybernetics, Rockville, MD, USA).

Immunohistochemistry

Sections were dewaxed with xylene and dehydrated with gradient alcohol. Sections were heated with 0.01 M citrate solution and allowed to cool down to room temperature after 20 min. After being maintained with 0.01 M PBS for 5 min, 3% hydrogen peroxide was added for 15 min to remove endogenous peroxidase. After PBS washing for 15 min, the sections were blocked with goat serum at 37°C for 20 min. The sections were incubated with primary rabbit anti-FOXK1 (1:1,000, ab18196; Abcam), rabbit anti-Snail (1:1,000, ab180714; Abcam), rabbit anti-MMP-2 (1:1,000, ab37150; Abcam), and rabbit anti-E-cadherin (1:500,000, ab76319; Abcam) overnight at 4°C. PBS served as NC instead of primary antibody. Sections were washed three times with PBS and incubated with goat anti-rabbit IgG (1:1,000, ab150117; Abcam) at 37°C for 30 min. Sections were rinsed with PBS for 15 min and incubated with streptavidin-biotin complex (Boster, Wuhan, P.R. China) at 37°C for 30 min. Color was developed by 3,3′-diaminobenzidine (DAB), followed by staining with hematoxylin for 1 min. Sections were washed with water for 1 min, followed by reaction in 1% hydrochloric acid and washing under running water. Sections were dehydrated and stained with saturated aluminum carbonate for 30 s, rinsed for 1 min, and treated with xylene for 15 min.

Tumor xenograft in nude mice

Twenty-four specific pathogen-free male BALB/c nude mice (4 weeks old, 18–25 g) were provided by Hunan SJA Laboratory Animal (Hunan, P.R. China). The stably transfected HuH-7 cells were resuspended in 10 mg/mL Matrigel (BD Biosciences, Bedford, MA, USA) at a volume ratio of 1:1. Cell concentration was adjusted to

5×10^6 cells/mL. Single-cell suspension (0.2 mL containing 1×10^6 cells) was subcutaneously injected into nude mice. Tumor size was monitored with a vernier caliper every week after 8 days of injection. Tumor volume (mm^3) was calculated by the formula: tumor volume = length $\times$ width^2 $\times$ 0.5. Six weeks after tumor cell inoculation, all nude mice were euthanized by carbon dioxide asphyxiation. Tumors were weighed.

Tumor metastatic model *in vivo*

Stably transfected HuH-7 cells (1×10^6 cells/mouse) were injected into nude mice via tail vein under sterile condition. Two weeks later, mice were euthanized by cervical dislocation to resect lung tissues. Under anatomy microscope, grayish white nodes were observed on the lung surface to be indicative of metastasis. Every five sites were selected for observation in each sample of lung tissues for statistical analysis.

Hematoxylin and eosin (H&E) staining

Lymph node tissues were fixed and then embedded in paraffin. Tissues were sliced to 4-μm-thick sections, dewaxed with xylene, hydrated with gradient ethanol, and washed with distilled water for 2 min. Sections were stained with hematoxylin for 5 min and rinsed in tap water. Sections were differentiated with hydrochloric acid ethanol for 30 s. Sections were then soaked in tap water for 15 min or warm water (about 50°C) for 5 min. Sections were then stained with eosin solution for 2 min. Sections were then treated with 95% ethanol (I) for 1 min, 95% ethanol (II) for 1 min, 100% ethanol (I) for 1 min, 100% ethanol (II) for 1 min, toluene carbonate (3:1) for 1 min, toluene (I) for 1 min, and xylene (II) for 1 min, in succession. Sections were sealed with resin and observed under an inverted microscope (XSP-8CA; Shanghai Optical Instrument, Shanghai, P.R. China).

Statistical analysis

SPSS 21.0 (IBM, Armonk, NY, USA) was employed for statistical analysis. Data were expressed as mean ± standard deviation. Data between two groups were compared using paired t test (cancer tissues and para-cancerous tissues) or unpaired t test. Data among multiple groups were compared by one-way analysis of variance (ANOVA) with Tukey's post hoc test. Data comparison between groups at different time points was performed using repeated-measures ANOVA with Bonferroni post hoc test. The correlation between SNHG1 and clinicopathological characteristics of patients with HCC was analyzed by chi-square test while correlation between two indicators was determined using Pearson's correlation analysis. The survival rate of patients was analyzed by Kaplan-Meier method. Log rank test was utilized for univariate analysis. Differences were considered statistically significant when $p < 0.05$.

SUPPLEMENTAL INFORMATION

Supplemental Information can be found online at https://doi.org/10.1016/j.omto.2021.02.002.

ACKNOWLEDGMENTS

This work was supported by the Traditional Chinese Medicine Science and Technology Development Plan Project of Shandong Province (nos. 2017-463 and 2019-0859), China Postdoctoral Science Foundation (nos. 2018M632679 and 2018M632687), and Key Research and Development Program of Shandong Province (no. 2019GSF108088). The authors would like to acknowledge the helpful comments on this paper received from the reviewers.

AUTHOR CONTRIBUTIONS

F.M. conceived and designed research. J.L. performed experiments. T.L. interpreted results of experiments. L.Z. analyzed data. J.W. prepared figures. Q.H. drafted the paper. A.Z. edited and revised the manuscript. All authors read and approved the final version of the manuscript.

DECLARATION OF INTERESTS

The authors declare no competing interests.

REFERENCES

1. Bray, F., Ferlay, J., Soerjomataram, I., Siegel, R.L., Torre, L.A., and Jemal, A. (2018). Global cancer statistics 2018: GLOBOCAN estimates of incidence and mortality worldwide for 36 cancers in 185 countries. CA Cancer J. Clin. *68*, 394–424.
2. Yang, J.D., Hainaut, P., Gores, G.J., Amadou, A., Plymoth, A., and Roberts, L.R. (2019). A global view of hepatocellular carcinoma: trends, risk, prevention and management. Nat. Rev. Gastroenterol. Hepatol. *16*, 589–604.
3. Sagnelli, E., Macera, M., Russo, A., Coppola, N., and Sagnelli, C. (2020). Epidemiological and etiological variations in hepatocellular carcinoma. Infection *48*, 7–17.
4. El-Serag, H.B. (2012). Epidemiology of viral hepatitis and hepatocellular carcinoma. Gastroenterology *142*, 1264–1273.e1.
5. Kulik, L., and El-Serag, H.B. (2019). Epidemiology and Management of Hepatocellular Carcinoma. Gastroenterology *156*, 477–491.e1.
6. Fujiwara, N., Friedman, S.L., Goossens, N., and Hoshida, Y. (2018). Risk factors and prevention of hepatocellular carcinoma in the era of precision medicine. J. Hepatol. *68*, 526–549.
7. Llovet, J.M., Montal, R., Sia, D., and Finn, R.S. (2018). Molecular therapies and precision medicine for hepatocellular carcinoma. Nat. Rev. Clin. Oncol. *15*, 599–616.
8. Parasramka, M.A., Maji, S., Matsuda, A., Yan, I.K., and Patel, T. (2016). Long noncoding RNAs as novel targets for therapy in hepatocellular carcinoma. Pharmacol. Ther. *161*, 67–78.
9. Huarte, M. (2015). The emerging role of lncRNAs in cancer. Nat. Med. *21*, 1253–1261.
10. Zheng, S., Li, M., Miao, K., and Xu, H. (2019). SNHG1 contributes to proliferation and invasion by regulating miR-382 in breast cancer. Cancer Manag. Res. *11*, 5589–5598.
11. Liu, Y., Yang, Y., Li, L., Liu, Y., Geng, P., Li, G., and Song, H. (2018). LncRNA SNHG1 enhances cell proliferation, migration, and invasion in cervical cancer. Biochem. Cell Biol. *96*, 38–43.
12. Li, Z., Lu, Q., Zhu, D., Han, Y., Zhou, X., and Ren, T. (2018). Lnc-SNHG1 may promote the progression of non-small cell lung cancer by acting as a sponge of miR-497. Biochem. Biophys. Res. Commun. *506*, 632–640.
13. Hu, Y., Ma, Z., He, Y., Liu, W., Su, Y., and Tang, Z. (2017). LncRNA-SNHG1 contributes to gastric cancer cell proliferation by regulating DNMT1. Biochem. Biophys. Res. Commun. *491*, 926–931.
14. Li, S.J., Wang, L., Sun, Z.X., Sun, S.J., Gao, J., and Ma, R.L. (2019). LncRNA SNHG1 promotes liver cancer development through inhibiting p53 expression via binding to DNMT1. Eur. Rev. Med. Pharmacol. Sci. *23*, 2768–2776.

15. Zhang, M., Wang, W., Li, T., Yu, X., Zhu, Y., Ding, F., Li, D., and Yang, T. (2016). Long noncoding RNA SNHG1 predicts a poor prognosis and promotes hepatocellular carcinoma tumorigenesis. Biomed. Pharmacother. *80*, 73–79.

16. Wang, Y., Cong, W., Wu, G., Ju, X., Li, Z., Duan, X., Wang, X., and Gao, H. (2018). MiR-376a suppresses the proliferation and invasion of non-small-cell lung cancer by targeting c-Myc. Cell Biol. Int. *42*, 25–33.

17. Yang, L., Wei, Q.M., Zhang, X.W., Sheng, Q., and Yan, X.T. (2017). MiR-376a promotion of proliferation and metastases in ovarian cancer: Potential role as a biomarker. Life Sci. *173*, 62–67.

18. Zheng, Y., Yin, L., Chen, H., Yang, S., Pan, C., Lu, S., Miao, M., and Jiao, B. (2012). miR-376a suppresses proliferation and induces apoptosis in hepatocellular carcinoma. FEBS Lett. *586*, 2396–2403.

19. Sukonina, V., Ma, H., Zhang, W., Bartesaghi, S., Subhash, S., Heglind, M., Foyn, H., Betz, M.J., Nilsson, D., Lidell, M.E., et al. (2019). FOXK1 and FOXK2 regulate aerobic glycolysis. Nature *566*, 279–283.

20. Wang, J., Liu, G., Liu, M., Xiang, L., Xiao, Y., Zhu, H., Wu, X., Peng, Y., Zhang, W., Jiang, P., et al. (2018). The FOXK1-CCDC43 Axis Promotes the Invasion and Metastasis of Colorectal Cancer Cells. Cell. Physiol. Biochem. *51*, 2547–2563.

21. Chen, F., Xiong, W., Dou, K., and Ran, Q. (2017). Knockdown of FOXK1 Suppresses Proliferation, Migration, and Invasion in Prostate Cancer Cells. Oncol. Res. *25*, 1261–1267.

22. Ma, X., Yang, X., Bao, W., Li, S., Liang, S., Sun, Y., Zhao, Y., Wang, J., and Zhao, C. (2018). Circular RNA circMAN2B2 facilitates lung cancer cell proliferation and invasion via miR-1275/FOXK1 axis. Biochem. Biophys. Res. Commun. *498*, 1009–1015.

23. Xu, H., Huang, S., Zhu, X., Zhang, W., and Zhang, X. (2018). FOXK1 promotes glioblastoma proliferation and metastasis through activation of Snail transcription. Exp. Ther. Med. *15*, 3108–3116.

24. Olmeda, D., Moreno-Bueno, G., Flores, J.M., Fabra, A., Portillo, F., and Cano, A. (2007). SNAI1 is required for tumor growth and lymph node metastasis of human breast carcinoma MDA-MB-231 cells. Cancer Res. *67*, 11721–11731.

25. Liu, K., Tang, Z., Huang, A., Chen, P., Liu, P., Yang, J., Lu, W., Liao, J., Sun, Y., Wen, S., et al. (2017). Glyceraldehyde-3-phosphate dehydrogenase promotes cancer growth and metastasis through upregulation of SNAIL expression. Int. J. Oncol. *50*, 252–262.

26. Tan, C., Cao, J., Chen, L., Xi, X., Wang, S., Zhu, Y., Yang, L., Ma, L., Wang, D., Yin, J., et al. (2019). Noncoding RNAs Serve as Diagnosis and Prognosis Biomarkers for Hepatocellular Carcinoma. Clin. Chem. *65*, 905–915.

27. Gao, S., Xu, X., Wang, Y., Zhang, W., and Wang, X. (2018). Diagnostic utility of plasma lncRNA small nucleolar RNA host gene 1 in patients with hepatocellular carcinoma. Mol. Med. Rep. *18*, 3305–3313.

28. Zhu, Q., Yang, H., Cheng, P., and Han, Q. (2019). Bioinformatic analysis of the prognostic value of the lncRNAs encoding snoRNAs in hepatocellular carcinoma. Biofactors *45*, 244–252.

29. Huang, D., Wei, Y., Zhu, J., and Wang, F. (2019). Long non-coding RNA SNHG1 functions as a competitive endogenous RNA to regulate PDCD4 expression by sponging miR-195-5p in hepatocellular carcinoma. Gene *714*, 143994.

30. Li, W., Dong, X., He, C., Tan, G., Li, Z., Zhai, B., Feng, J., Jiang, X., Liu, C., Jiang, H., and Sun, X. (2019). LncRNA SNHG1 contributes to sorafenib resistance by activating the Akt pathway and is positively regulated by miR-21 in hepatocellular carcinoma cells. J. Exp. Clin. Cancer Res. *38*, 183.

31. Zhang, H., Zhou, D., Ying, M., Chen, M., Chen, P., Chen, Z., and Zhang, F. (2016). Expression of Long Non-Coding RNA (lncRNA) Small Nucleolar RNA Host Gene 1 (SNHG1) Exacerbates Hepatocellular Carcinoma Through Suppressing miR-195. Med. Sci. Monit. *22*, 4820–4829.

32. Zhang, S., and Song, X. (2019). Long non-coding RNA SNHG1 promotes cell proliferation and invasion of hepatocellular carcinoma by acting as a molecular sponge to modulate miR-195. Arch. Med. Sci. *16*, 386–394.

33. He, H., Chen, D., Cui, S., Piao, H., Tang, H., Wang, X., Ye, P., and Jin, S. (2019). Identification of a long non-coding RNA-mediated competitive endogenous RNA network in hepatocellular carcinoma. Oncol. Rep. *42*, 745–752.

34. Liu, J., Li, W., Zhang, J., Ma, Z., Wu, X., and Tang, L. (2019). Identification of key genes and long non-coding RNA associated ceRNA networks in hepatocellular carcinoma. PeerJ *7*, e8021.

35. Hu, Y., Sun, L., Tao, S., Dai, M., Wang, Y., Li, Y., and Wu, J. (2019). Clinical significance of HDAC9 in hepatocellular carcinoma. Cell. Mol. Biol. *65*, 23–28.

36. Li, P., Yu, Z., He, L., Zhou, D., Xie, S., Hou, H., and Geng, X. (2017). Knockdown of FOXK1 inhibited the proliferation, migration and invasion in hepatocellular carcinoma cells. Biomed. Pharmacother. *92*, 270–276.

37. Cao, H., Chu, X., Wang, Z., Guo, C., Shao, S., Xiao, J., Zheng, J., and Zhang, D. (2019). High FOXK1 expression correlates with poor outcomes in hepatocellular carcinoma and regulates stemness of hepatocellular carcinoma cells. Life Sci. *228*, 128–134.

38. Cui, H., Gao, Q., Zhang, L., Han, F., and Wang, L. (2018). Knockdown of FOXK1 suppresses liver cancer cell viability by inhibiting glycolysis. Life Sci. *213*, 66–73.

39. Liu, J., Wu, Z., Han, D., Wei, C., Liang, Y., Jiang, T., Chen, L., Sha, M., Cao, Y., Huang, F., et al. (2020). Mesencephalic Astrocyte-Derived Neurotrophic Factor Inhibits Liver Cancer Through Small Ubiquitin-Related Modifier (SUMO)ylation-Related Suppression of NF-κB/Snail Signaling Pathway and Epithelial-Mesenchymal Transition. Hepatology *71*, 1262–1278.

40. Huang, Q., Pu, M., Zhao, G., Dai, B., Bian, Z., Tang, H., Chen, C., Liu, W., Qu, X., Shen, L., and Tao, K. (2017). Tg737 regulates epithelial-mesenchymal transition and cancer stem cell properties via a negative feedback circuit between Snail and HNF4α during liver stem cell malignant transformation. Cancer Lett. *402*, 52–60.

41. Peng, Y., Zhang, P., Huang, X., Yan, Q., Wu, M., Xie, R., Wu, Y., Zhang, M., Nan, Q., Zhao, J., et al. (2016). Direct regulation of FOXK1 by C-jun promotes proliferation, invasion and metastasis in gastric cancer cells. Cell Death Dis. *7*, e2480.

42. Guo, X., and Wang, Y. (2020). LncRNA TMPO-AS1 promotes hepatocellular carcinoma cell proliferation, migration and invasion through sponging miR-329-3p to stimulate FOXK1-mediated AKT/mTOR signaling pathway. Cancer Med. *9*, 5235–5246.

OMTO, Volume *21*

Supplemental information

SNHG1 knockdown upregulates miR-376a and downregulates FOXK1/Snail axis to prevent tumor growth and metastasis in HCC

Fanzhi Meng, Jinghua Liu, Tao Lu, Lanlan Zang, Jing Wang, Qiang He, and Aijin Zhou

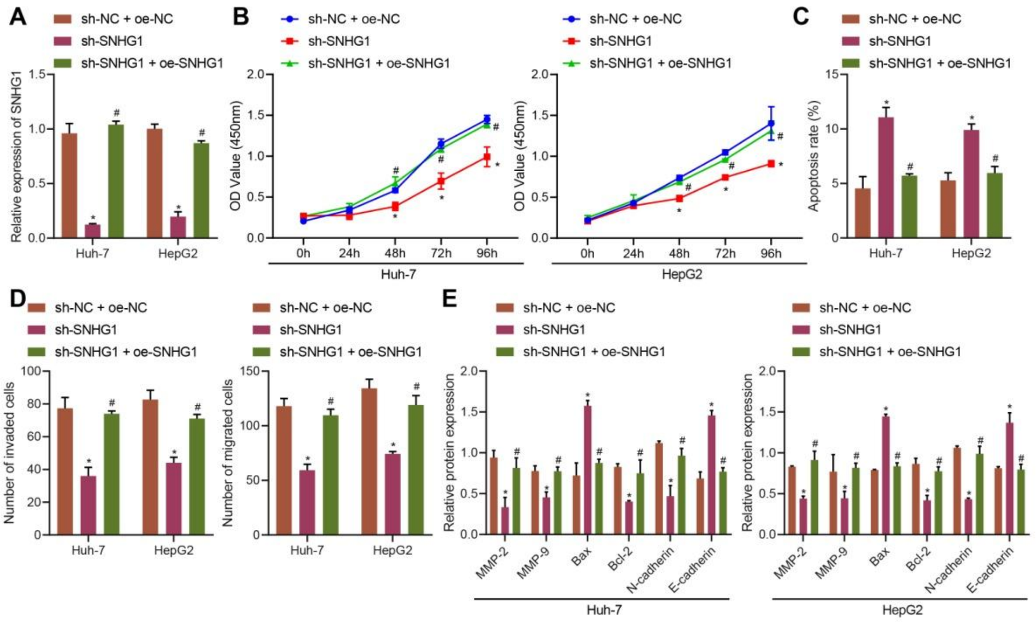

Supplementary Figure 1 Effect of downregulated SNHG1 on HCC cell phenotypes is rescued by overexpressed SNHG1. A: SNHG1 expression determined by RT-qPCR; B: Cell viability assessed by CCK8 assay; C: Cell apoptosis determined by flow cytometry; D: Cell invasion and migration determined by Transwell assay; E: Expression of MMP-2, MMP-9, Bax, Bcl-2, E-cadherin and N-cadherin proteins measured by Western blot assay; * $p < 0.05$ *vs*. cells co-transfected with sh-NC and oe-NC; # $p < 0.05$ *vs*. cells co-transfected with sh-SNHG1. Data were expressed as mean ± standard deviation. Data between two groups were compared with unpaired *t* test. Data among multiple groups were compared with one-way ANOVA with Tukey's post hoc test. Data comparison among groups at different time points was performed using repeated measures ANOVA and Bonferroni post hoc test. Each experiment was repeated 3 times independently.

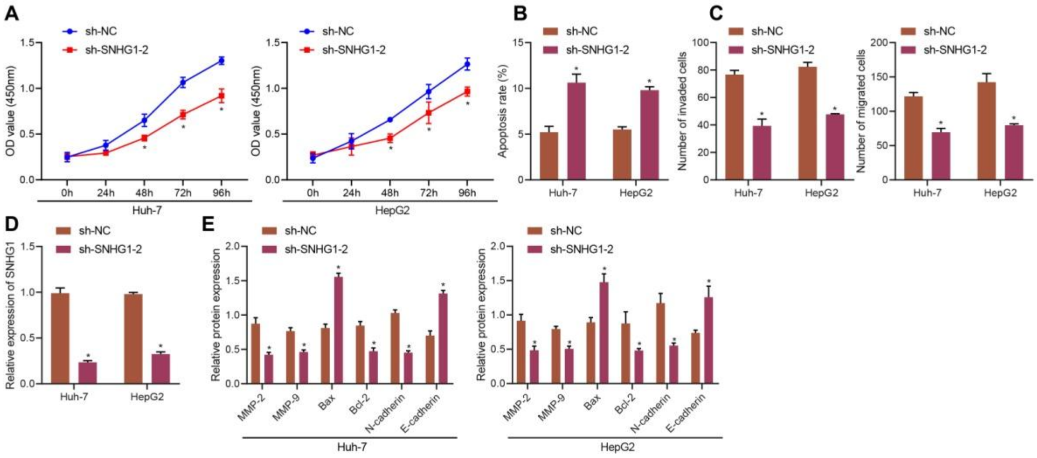

Supplementary Figure 2 sh-SNHG1-2 effectively reduces SNHG1 expression to mediate HCC cell phenotypes and related protein expression. A: Cell viability assessed by CCK8 assay; B: Cell apoptosis determined by flow cytometry; C: Cell invasion and migration determined by Transwell assay; D: SNHG1 expression determined by RT-qPCR; E: Expression of MMP-2, MMP-9, Bax, Bcl-2, E-cadherin and N-cadherin proteins measured by Western blot assay; * $p < 0.05$ *vs*. cells transfected with sh-NC. Data were expressed as mean ± standard deviation. Data between two groups were compared with unpaired *t* test. Data among multiple groups were compared with one-way ANOVA with Tukey's post hoc test. Data comparison among groups at different time points was performed using repeated measures ANOVA and Bonferroni post hoc test. Each experiment was repeated 3 times independently.

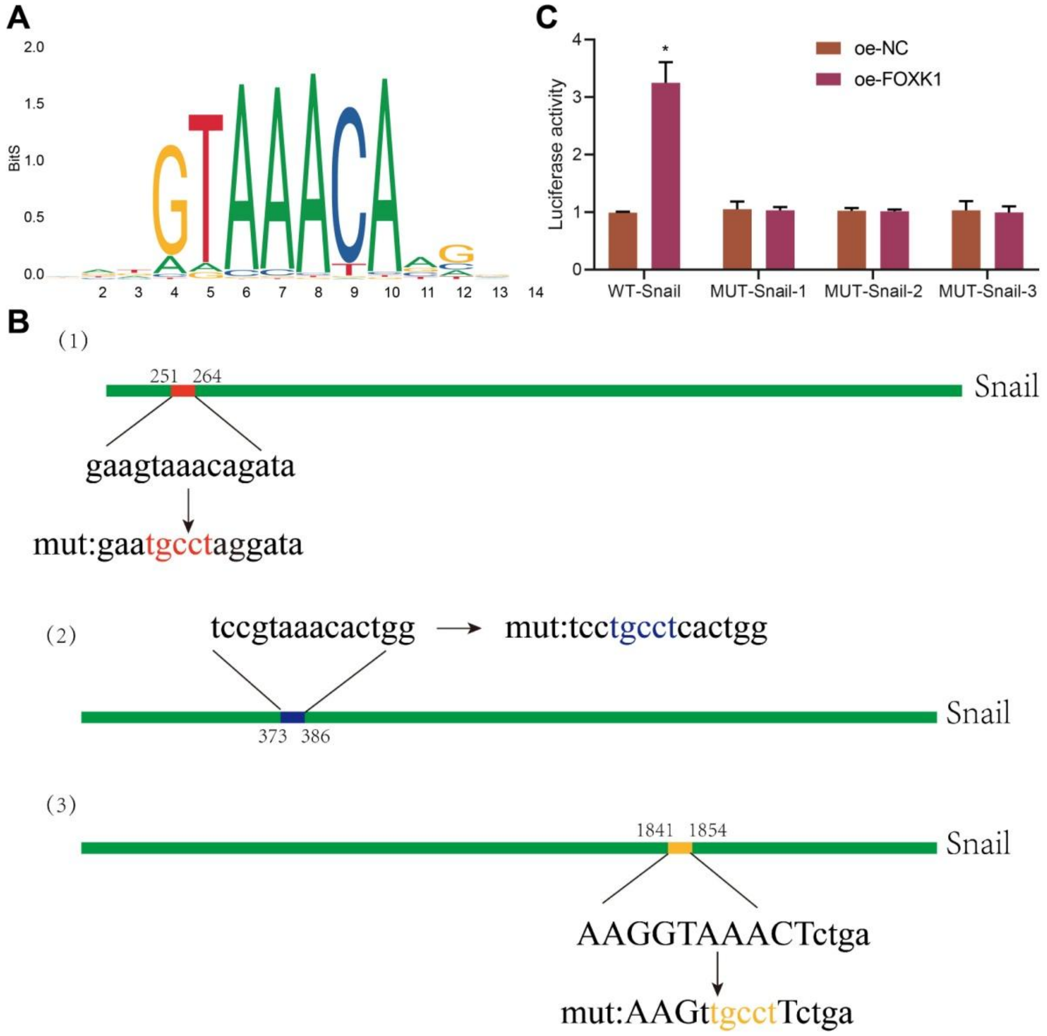

Supplementary Figure 3 Prediction results of binding sites between FOXK1 and Snail promoter region. A: Matrix model for prediction on binding motifs between FOXK1 and Snail; B: Binding sites between FOXK1 and Snail and the corresponding mutation.
